# Large dosage Huangqin (Scutellaria) and Huanglian (Rhizoma Coptidis) for T2DM: A protocol of systematic review and meta-analysis of randomized clinical trials

**DOI:** 10.1097/MD.0000000000022032

**Published:** 2020-09-18

**Authors:** Xiaoying Huang, Lizhen Wang, Rensong Yue, Ning Ding, Hongjing Yang

**Affiliations:** a Hospital of Chengdu University of Traditional Chinese Medicine; b Chengdu University of Traditional Chinese Medicine, Chengdu, Sichuan, China.

**Keywords:** huanglian (rhizoma coptidis), huangqin (scutellaria), large dosage, meta-analysis and systematic review, protocol, T2DM

## Abstract

**Introduction:**

Type 2 diabetes mellitus (T2DM) is a metabolic disease with widespread concern in the world. It has the characteristics of high incidence rate and high disability rate, which seriously affects economic and social development. large dose herb Rhizoma Coptidis (Huanglian) and Scutellaria (Huangqin) or compound prescription contain large dose Huanglian and Huanglian for treatment of T2DM has already been confirmed. However, due to the lack of evidence, there is no specific method or suggestion, so it is necessary to carry out systematic evaluation on Coptidis and Scutellaria and provide effective evidence for further research.

**Methods and analysis:**

The following databases will be searched from their inception to June 2020: Electronic database includes PubMed, Embase, Cochrane Library, Web of Science, Nature, Science online, Chinese Biomedical Database WanFang, VIP medicine information, and China National Knowledge Infrastructure. Primary outcomes: fasting blood-glucose (FBG), 2 Hours Postprandial Blood Glucose (2hPBG), Glycosylated hemoglobin A1c (HbA1c). additional outcomes: Low Density Lipoprotein (LDL), High Density Lipoprotein (HDL), triglycerides (TG), total serum cholesterol (TC). Data will be extracted by 2 researchers independently, risk of bias of the meta-analysis will be evaluated based on the Cochrane Handbook for Systematic Reviews of Interventions. All data analysis will be conducted by data statistics software Review Manager V.5.3. and Stata V.12.0.

**Results:**

The results of this study will systematically evaluate the effectiveness and safety of large dose Huanglian and Huangqin intervention for people with T2DM.

**Conclusion:**

The systematic review of this study will summarize the current published evidence of large dose Huanglian and Huangqin for the treatment of T2DM, which can further guide the promotion and application of it.

**Ethics and dissemination:**

This study is a systematic review, the outcomes are based on the published evidence, so examination and agreement by the ethics committee are not required in this study. We intend to publish the study results in a journal or conference presentations.

**Open Science Framework (OSF) registration number:**

July 21, 2020. osf.io/b6r3z. (https://osf.io/b6r3z)

## 1 Introduction

Type 2 diabetes (T2DM) is a metabolic disease characterized by impaired islet β-cell function and insulin resistance.^[1]^ In recent years, its incidence has increased year by year due to changes of living environment and lifestyle. According to the International Diabetes Federation (IDF) 2019 9th Edition diabetes map, about 463 million people worldwide have diabetes.^[2]^ It respectively costed $294.6 billion and $109 billion a year to face diabetic health problems in the United States and China.^[3,4]^

In the treatment, Early symptoms can be relieved by exercise and diet.^[5]^ Also, blood sugar can be controlled by taking insulin sensitizer or insulin secretagogue When the damage of islet function is not serious.^[3]^ finally, insulin should be taken when the islet function is severely damaged.^[3]^ However, both the last 2 treatments will lead to many side effects, such as hypoglycemia, nausea and vomiting,^[6]^ which will seriously affect the life quality of patients.

In recent years, traditional Chinese medicine has been widely used in clinical and experimental study of T2DM, which had been fully proven effective. Rhizoma Coptidis (Huanglian) and Scutellaria (Huangqin) are botanical herbs commonly used in traditional Chinese medicine and the exploration of diabetes treatment methods show that large-dose Huanglian and Huangqin had good effects on the controlling of blood sugar and improvement of diabetes symptoms,^[7]^ but its effectiveness and safety have not yet reached a definitive conclusion. Therefore, this research intends to adopt the method of system valuation and meta-analysis of large-dose Huanglian and Huangqin or contain large-dose Huanglian and Huangqin formulae in the treatment of type 2 diabetes to evaluate the efficacy and safety.

## 2 Methods

### 2.1 Study registration

The protocol has been registered in OSF (Open Science Framework) Preregistration. July 21,2020. osf.io/b6r3z.(https://osf.io/b6r3z). The protocol will follow the statement guidelines of Preferred Reporting Items for Systematic Reviews and Meta-Analyses Protocols (PRISMAP),^[8]^ Changes will be reported in the full review as required.

### 2.2 Inclusion and exclusion criteria for study selection

#### 2.2.1 Inclusion criteria

Inclusion criteria are all randomized controlled trials (RCTs), Which treatment of T2DM are large dose Huanglian and Huangqin serves as 2 herbs or the 2 herbs are main elements in mixture herb formulas. The language of the trials to be included only Chinese or English.

#### 2.2.2 Exclusion criteria. Following stuides will be excluded

1. patients age <18 years old
2. other types of diabetes. Such as Type 1 diabetes, Gestational diabetes and so on.
3. the treatment was combined with other treatment other than Chinese herbs.
4. Non-RCTs and Quasi-RCTs
5. Case series and Reviews
6. Animal studies

### 2.3 Types of participants

Types of participants included people diagnosed with T2DM, no matter the degree and possible complications. All the patients should be treated by traditional Chinese medicine included 2 herbs huangqian and huanglia, or the 2 herbs are main elements in mixture herb formulas, or the 2 herbs combine with other conventional treatments. No sex, ethnicity, or education restriction is here.

### 2.4 Experimental interventions

The traditional Chinese medicine Huanglian and Huanglian should be the main treatments. Dosage limits of these 2 herbs should be: large dose Huanglian (more than 20 g, 4 times larger than the prescribed maximum dose of *Chinese Pharmacopeia* 2010 version), large does Huangqin (more than 20 g, 2 times larger than the prescribed maximum dose of *Chinese Pharmacopeia* 2010 version).

### 2.5 Control interventions

Interventions may include: No treatment, The placebo, Non-drug interventions (eg, diet, exercise, etc), Conventional western medicine hypoglycemic drugs (eg, metformin, euglycemic, etc), Insulin (any kind of insulin). Combined interventions are allowed if all groups in the randomized trial receive the same combined intervention.

### 2.6 Types of outcome measures

#### 2.6.1 Main outcomes

(1) Glycosylated hemoglobin A1c (HbA1c);
(2) fasting blood-glucose (FBG);
(3) 2 Hours Postprandial Blood Glucose (2hPBG).

#### 2.6.2 Additional outcomes

(1) Low Density Lipoprotein (LDL)
(2) High Density Lipoprotein (HDL)
(3) triglycerides (TG)
(4) total serum cholesterol (TC).

## 3 Data sources

### 3.1 Electronic searches

The following data bases will be searched to identify eligible studies: PubMed, Embase, Cochrane Library, Web of Science, Nature, Science on line, Chinese Biomedical Database WanFang, VIP medicine information, and China National Knowledge Infrastructure. The time range is: the starting time is determined according to the first literature available, and the deadline is June 2020.

### 3.2 Other search resources

**Figure 1:**
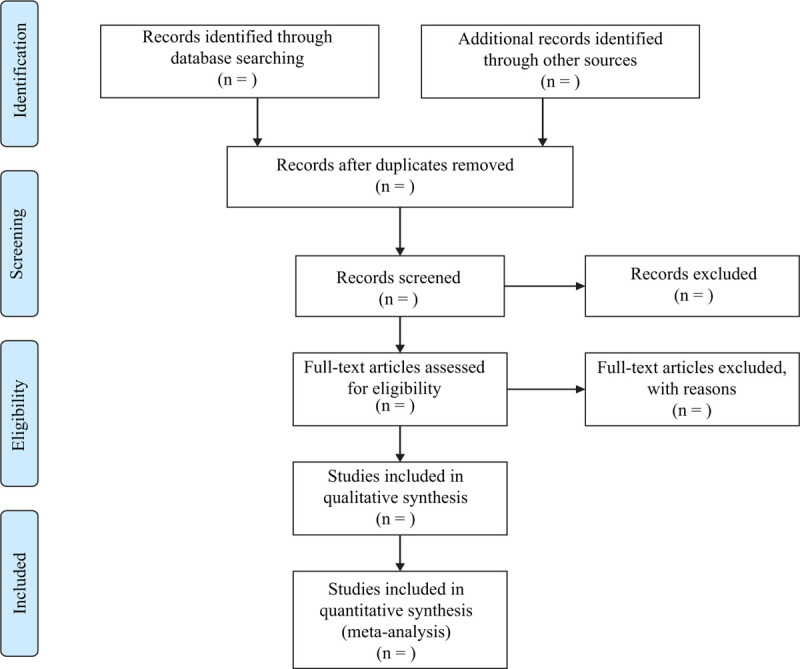
The research flowchart.

In order to get more complete evidence, we will also retrieve other related documents by manually, such as medical textbooks, clinical laboratory manuals and so on. If it is necessary to contact with trail author to obtain the latest clinical data, we will do it. Moreover, studies associated with the review will be identified via evaluating related conference proceedings. The research flowchart is shown in Figure 1.

### 3.3 Search strategy

The following search terms will be used: RCT; type 2 diabetes/T2DM; traditional chinese medicine/TCM; huang-lian/Huanglian/coptis, huang-qin/Huangqin/Scutellaria. different retrieval strategies in Chinese and foreign databases will be used. Language restrictions are Chinese and English. There is no publication restriction. Here we take the search strategy in PubMed as an example and list in Table 1.

**Table 1:**
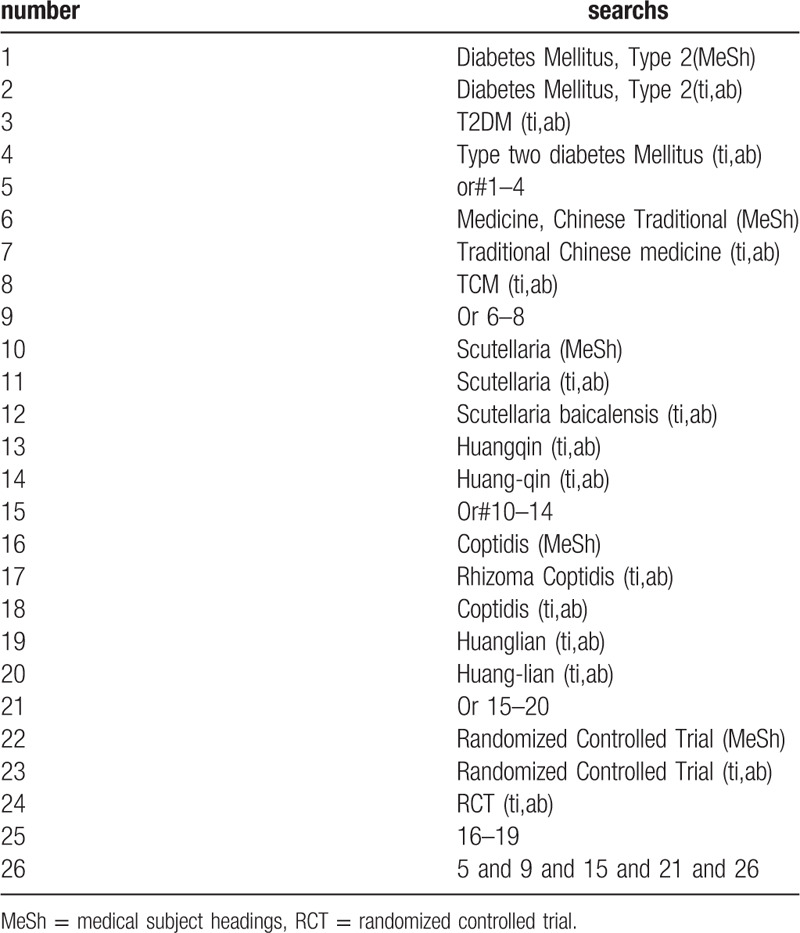
Search stragtegy sample of PubMed.

## 4 Data collection and analysis

### 4.1 Study selection

All articles in the search results were independently evaluated by 2 independent researchers (HX, WL) according to inclusion and exclusion criteria. Reviewers will then independently extract and collect the data included in the study using pre-designed data collection forms. Discrepancies will be discussed and resolved by consensus with a third author (YR).

### 4.2 Data extraction and management

The following information will be extracted from each study

(1) Normal test characteristics: title, author, year.
(2) baseline data: sample size, age, gender, diagnostic criteria, course of disease.
(3) Interventions: dosage of Huanglian and Huangqin, control of intervention details, intervention. If the information is not enough, we will contact experts and authors in this field to get relevant information.

### 4.3 Assessment of the reporting quality and risk of bias

The risk of bias will be assessed by 2 independent authors (HX and WL), together with completing the STRICTA checklist.^[9]^ The Cochrane System Evaluator's Manual give the evaluation criteria for authors to evaluated the RCTs’ quality. Assessing the risk of bias:

(1) random sequence generation;
(2) allocation concealment;
(3) blinding of participants and personnel;
(4) blinding of outcome assessment;
(5) incomplete outcome data;
(6) selective outcome reporting;
(7) other bias.

Any disagreement will be discussed or consulted with a third reviewer. Each them will be described from 3 levels: “high risk”, “low risk” and “unclear”.

### 4.4 Measures of a treatment effect

The dichotomous outcomes will be expressed by the Odds ratio (ORs), while the continuous data will use the Standardized mean difference (SMD). All these outcomes report 95% confidence interval.

### 4.5 Management of missing data

We will take the method of contacting corresponding authors to obtain the missing data. If there is no response, we will analyze only the available data and describe the reason and impact of this exclusion in the paper.

### 4.6 Assessment of a reporting bias

The bias of publication will be explored through funnel plot analysis. If the funnel plot show asymmetry, it will be evaluated via the Egger and Beg tests, and *P* value < .05 means the publication bias is significant.

### 4.7 Assessment of heterogeneity

There are 2 main methods for testing heterogeneity, namely graphical method (funnel plot, forest plot) and statistical test (*Q* value statistic test, *I*^2^ statistic test, *H* statistic test). The *I*^2^ statistic test method shows us When *I*^2^ is 0, it means that studies are completely homogeneous, If *I*^2^ > 50%, it indicates there is heterogeneity in studies.

### 4.8 Data synthesis and grading of quality of evidence

The results of the study will be analyzed by RevMan 5.0 software provided by Cochrane collaborate on network. The binary data will be expressed by the odds ratio, while the continuous data will use the mean difference (MD). To test the heterogeneity of the research results, when the *I*^2^ < 50% or *P* > .1, the heterogeneity is significant. The random effect model was used for the meta-analysis, otherwise, we choose the fixed effect model.

### 4.9 Subgroup analysis

When the heterogeneity test results are heterogeneous, we need to clarify the source of the heterogeneity by subgroup analysis. The effects of different types of therapy including design scheme, severity of illness, age, sex, and mild or severe T2DM were analyzed. We will also delete low-quality and/or medium-quality studies to check the robustness of the results.

### 4.10 Sensitivity analysis

Sensitivity analysis can not only assess the stability and reliability of the conclusions of the Meta-analysis, but also assess whether the changes in the results are related to the impact of a single study. If the stability of the conclusion is poor, we can achieve the purpose of increasing stability by changing the analysis model, inclusion criteria and exclusion criteria, or excluding a certain type of literature.

### 4.11 Ethics and dissemination

We will publish the system review results in peer-reviewed journals, disseminated in meetings or in peer-reviewed publications. Aggregated published data will be used to excluded data of individuals, so there is no need for obtaining the ethical approval or patients’ informed consent.

## 5 Discussion

In traditional Chinese medicine, Coptidis and Scutellaria are commonly used herbs with it efficacy of clearing heat-toxin. They can be used for a variety of metabolic diseases such as obesity, hyperlipidemia, coronary heart disease, and type 2 diabetes mellitus. Their effectiveness in the treatment of T2DM is confirmed from the following aspects:

(1) improve damaged islet cells,^[10]^
(2) perfect the state of insulin resistance,^[11]^
(3) improve sugar and blood lipid disorders,^[12]^
(4) improve long-standing chronic metabolic state of inflammation in patients,^[13]^
(5) improve the imbalance of intestinal flora, increase the beneficial bacterium group and against bacteria inhibition.^[14]^

In addition, clinical experiments have also confirmed that a variety of compound Traditional Chinese formulas contains Scutellaria and Coptidis such as Gegenqinlian decoction and Pinellia-Xiexin decoction can significantly improve the clinical symptoms and serum biochemical indexes of T2DM,^[15,16]^ achieving the goal of blood glucose control and improve the patients’ quality of life.

The grateful effectiveness of Scutellaria and Coptidis for T2DM may be related to the main active ingredients.

Coptidis mainly includes berberine, rhizoma coptidis, epiberberine, and other biologically active bases.^[17]^ Berberine is the main pharmacological function composition, and its resistance to T2DM mainly lies in following characteristics^[18,19]^:

(1) improve the state of T2DM chronic inflammation,
(2) activate brown adipose tissue or stimulus brown adipose tissue change to white adipose tissue to promote energy metabolism,
(3) improve glucose metabolism and insulin resistance, improve the function of the islet beta cells secrete insulin.

However, some studies have also shown that berberine is the most important toxic component of coptis alkaloids, its mortality and morbidity have not been clearly defined in clinical and animal experiments, so its toxicological mechanism and further study are still needed.

The chemical constituents of Scutellaria are flavonoids, mainly include baicalin, etc. Scutellaria has definite pharmacological effects such as anti-inflammatory, antiviral, antioxidant and treatment of diabetes.^[20]^ For example, Baicalin can reduce the inflammatory response of pancreatic beta cells and inhibit the process of apoptosis. This change is related to the regulation of mirNA-205.^[21]^ It can also alleviate insulin resistance in obese rats by accelerating the translocation of Glut-4.^[22]^ In addition, a variety of chemical components in Scutellaria have inhibitory effects on-glucosidase and -amylase activities.^[23]^

Doctors of traditional Chinese medicine has always been refined to consider the herbs’ dosage. In terms of the dosage of Coptis, the standard in the Chinese Pharmacopoeia is 2 to 5 g, while Scutellariae is 3 to 10 g. However, in clinical practices, Chinese physicians have their own unique cognition. In terms of the ratio, Cui Xiang^[24]^ proved that when the ratio of scutellaria to Coptis was 3:2 to 1:3, both them had significant synergistic effect. While the ratios turn to 1:1, they had strongest synergistic effect than any other ratios. In terms of specific dosage, Academician Tong Xiaolin^[25]^ proposed that the treatment of T2DM by Huangqin and Huanglian should follow the principle of “Serious diseases need to be treated with violent herbs”, the dosage of Scutellaria should be 9 to 15 g while Coptis should be 9 to 30 g, sometimes the dosage of Coptis can reach 15 to 30 g. The reason using large dosage to reach treatment effect is that Traditional Chinese medicine is rare play the effect by single herbs, commonly by the form of compound herbs or prescriptions. Clinical experience confirmed the dosage of coptidis and its compound is generally larger. Therefore, it is also suggested that it is necessary to consider Chinese herbs rather than chemical extracts in clinical medication, and the dosage should be more in line with clinical practice, to accurately reflect the functional value of traditional Chinese medicine and its compound.

In conclusion, the systematic review and meta-analysis are helpful to determine the potential value of high-dose Scutellaria and coptidis and the 2 herbs combination therapy for T2DM. To improve the quality of life in severe patients. This study can not only provide the basis for the release of diabetes treatment guidelines, but also promote the application of traditional Chinese medicine prescriptions, so that more patients benefit.

## Author contributions

**Conceptualization**: Lizhen Wang, Xiaoying Huang, Rensong Yue.

**Data curation**: Hongjing Yang; Ning Ding.

**Formal analysis**: Lizhen Wang, Xiaoying Huang.

**Methodology**: Lizhen Wang, Xiaoying Huang, Rensong Yue.

**Project administration**: Rensong Yue.

**Resources**: Lizhen Wang, Xiaoying Huang, Hongjing Yang.

**Software**: Lizhen Wang, Xiaoying Huang.

**Supervision**: Rensong Yue.

**Writing – original draft**: Lizhen Wang

**Writing – review & editing**: Rensong Yue.

## Abbreviations

Abbreviations: Huanglian = Rhizoma Coptidis, Huangqin = Scutellaria, RCT = randomized controlled trial.
